# Massively HIV-1-infected macrophages exhibit a severely hampered ability to differentiate into osteoclasts

**DOI:** 10.3389/fimmu.2023.1206099

**Published:** 2023-06-19

**Authors:** Franco A. Sviercz, Patricio Jarmoluk, Cintia G. Cevallos, Cynthia A. M. López, Rosa N. Freiberger, Alex Guano, Alan Adamczyk, Matías Ostrowski, M. Victoria Delpino, Jorge Quarleri

**Affiliations:** Instituto de Investigaciones Biomédicas en Retrovirus y Sida (INBIRS); Universidad de Buenos Aires, Consejo Nacional de Investigaciones Científicas y Técnicas (CONICET), Buenos Aires, Argentina

**Keywords:** HIV, osteoclast, macrophage, bone, TRAP

## Abstract

**Introduction:**

Osteoclasts play a crucial role in bone resorption, and impairment of their differentiation can have significant implications for bone density, especially in individuals with HIV who may be at risk of altered bone health. The present study aimed to investigate the effects of HIV infection on osteoclast differentiation using primary human monocyte-derived macrophages as precursors. The study focused on assessing the impact of HIV infection on cellular adhesion, cathepsin K expression, resorptive activity, cytokine production, expression of co-receptors, and transcriptional regulation of key factors involved in osteoclastogenesis.

**Methods:**

Primary human monocyte-derived macrophages were utilized as precursors for osteoclast differentiation. These precursors were infected with HIV, and the effects of different inoculum sizes and kinetics of viral replication were analyzed. Subsequently, osteoclastogenesis was evaluated by measuring cellular adhesion, cathepsin K expression, and resorptive activity. Furthermore, cytokine production was assessed by monitoring the production of IL-1β, RANK-L, and osteoclasts. The expression levels of co-receptors CCR5, CD9, and CD81 were measured before and after infection with HIV. The transcriptional levels of key factors for osteoclastogenesis (RANK, NFATc1, and DC-STAMP) were examined following HIV infection.

**Results:**

Rapid, massive, and productive HIV infection severely impaired osteoclast differentiation, leading to compromised cellular adhesion, cathepsin K expression, and resorptive activity. HIV infection resulted in an earlier production of IL-1β concurrent with RANK-L, thereby suppressing osteoclast production. Infection with a high inoculum of HIV increased the expression of the co-receptor CCR5, as well as the tetraspanins CD9 and CD81, which correlated with deficient osteoclastogenesis. Massive HIV infection of osteoclast precursors affected the transcriptional levels of key factors involved in osteoclastogenesis, including RANK, NFATc1, and DC-STAMP.

**Conclusions:**

The effects of HIV infection on osteoclast precursors were found to be dependent on the size of the inoculum and the kinetics of viral replication. These findings underscore the importance of understanding the underlying mechanisms to develop novel strategies for the prevention and treatment of bone disorders in individuals with HIV.

## 1 Introduction

Patients with Human Immunodeficiency Virus type 1 (HIV) infection frequently have low bone density, thus increasing significantly their risk of fractures (1). Antiretroviral medication has considerably extended patient survival, but long-term side effects such as bone abnormalities have also emerged. Bone loss in infected people is caused by a variety of variables, including antiretroviral medication, patient lifestyle, and health (such as age, body mass index, or muscle wasting) (2, 3). Additionally, bone deficiencies in individuals who are not receiving treatment point to the virus’s independent involvement (4–7). Due to the activity of the bone-resorbing osteoclasts, the bone-forming osteoblasts, and the osteocytes, the skeleton is a dynamic organ that is constantly remodeling. Bone flaws can result from an imbalance between osteoclasts and osteoblasts. HIV proteins such as gp120, Tat, and Nef have been shown to have impacts on bone cells, including osteoblasts, in several *in vitro* investigations (8–10). However, bone loss in HIV-infected individuals is linked to an increase in blood indicators of bone resorption but not or only little altered markers of bone growth, indicating a significant role for osteoclasts (7). While osteoclasts form primarily through the fusion of monocyte/macrophage precursors derived from hematopoietic stem cells, osteoblasts emerge from cells of mesenchymal origin (11, 12).

Macrophage Colony-Stimulating Factor (M-CSF) and Receptor Activator of Nuclear Factor-kappa B Ligand (RANK-L) regulate the differentiation of myeloid progenitors into osteoclasts as multinucleated gigantic cells (13). The level of osteoclasts differentiation is primarily influenced by RANK-L activity, which is restrained by its physiological decoy receptor osteoprotegerin (OPG) (14, 15). The transcription factor Nuclear Factor of Activated T Cells Cytoplasmic 1 (NFATc1) is primarily activated during osteoclasts differentiation (16). Osteoclasts are multinucleated cells with the rare capacity to resorb bone matrix. They are derived from the monocytic lineage and produced by the fusion of mononucleated precursors, such as blood-circulating monocytes and bone-resident precursors, to undergo final differentiation (12). Additionally, osteoclastogenesis, or the recruitment and/or differentiation of osteoclasts precursors, is favored by proinflammatory cytokines such as tumor necrosis factor (TNF)-α, interleukin (IL)-6, and IL-1β, which create a favorable environment for osteoclastogenesis (17). The v3 integrin adhesion receptor, tartrate-resistant acid phosphatase (TRAP), and resorption-related enzymes such as cathepsin K are all highly expressed in terminally differentiated osteoclasts. Only a few mechanisms, including increased production of proinflammatory cytokines (18), immune system disruption (7, 19–23), and stimulation of the bone resorption activity of infected osteoclasts (24–26), have been put forward to explain the rise in osteolytic activity linked to HIV infection. HIV infection increases the RANK-L/OPG ratio, which drives osteoclasts differentiation via acting on T and B cells (27).

As HIV-essential host cells, macrophages are able to maintain active viral replication *in vivo*, with higher resistance to the cytopathic effects of the virus than CD4+ T cells. Macrophages are distributed throughout the majority of the organism’s tissues, thus acting as ubiquitous HIV reservoirs and a means of viral transmission and dissemination (28–33). A direct impact of HIV infection on macrophages may support the observed *in vitro* acquisition of some osteoclasts characteristics (multinucleation, enhanced ability to degrade organic matrices, and organization of their podosomes into circular structures when seeded on glass) (32, 34). Additionally, it has been demonstrated that HIV affects mature osteoclasts, causing changes to the structure and functionality of the sealing zone, as well as osteoclasts precursors, promoting their migration to bones and differentiation. The ability for bone disintegration and improved osteoclasts adherence is significantly correlated with these alterations. In this context, the C-C chemokine receptor type 5 (CCR5) expressed on macrophages is used by R5-tropic HIV strains for its cellular entry and appears to play essential roles in bone-destructive conditions through the functional regulation of osteoclasts since its blockade impairs osteoclasts differentiation and their functional cellular architecture through regulating integrin-and chemokine-mediated pathways (35). Besides, other consequences derived from HIV-infected macrophages can impact the microenvironment of osteoclast precursors recruitment or differentiation through changes in secreted soluble mediators. The HIV infection kinetics in macrophages differs according to viral and host-related factors. Among the former, viral inoculum and viral strain are involved (36, 37).

Here, we investigated whether macrophages -as osteoclasts precursors- might be reprogrammed differentially toward osteoclasts as a result of the magnitude of the viral inoculum, and its consequences on the macrophage phenotype in order to better understand the processes underlying the bone abnormalities brought on by HIV infection.

## 2 Materials and methods

### 2.1 Monocyte-derived macrophages culture and differentiation to osteoclasts

Primary human monocytes were isolated from the blood of healthy donors and differentiated as monocyte-derived macrophages (MDM), as described previously (38). The purity of the isolated CD14+ monocytes was more than 80% as determined by flow cytometry. Briefly, monocytes were seeded on slides in 24-well plates at a density of 5 × 10^5^ cells/mL in Roswell Park Memorial Institute medium (RPMI, Gibco, Grand Island, NY, USA) supplemented with 10% FBS, 2 mM of L-glutamine (Gibco), 1 mM of sodium pyruvate (Gibco), penicillin-streptomycin and M-CSF (30 ng/mL) (StemCell Technologies, Canada) for 6 days (osteoclast precursors). Then, mature OC were obtained from cultured MDM (osteoclast precursors) in alpha minimum essential medium (α-MEM) (Gibco) supplemented with 10% FBS, 2 mM of L-glutamine (Gibco), 1 mM of sodium pyruvate (Gibco), and penicillin-streptomycin, M-CSF (30 ng/mL) and RANKL (50 ng/mL) (both from StemCell Technologies, Canada) for 9 days (mature osteoclast).

The studies performed in this work have been reviewed and approved by the institutional review board and local ethical committee. Buffy coats from healthy donors, between 18 and 60 years old and a balanced female: male ratio, were obtained from Hospital de Clínicas ‘José de San Martín’, Facultad de Medicina, Universidad de Buenos Aires. All human samples used in this study would have been obtained even if this study was not carried out, and were supplied without any personally identifiable information.

### 2.2 Cell-Free wild type-HIV and pseudotyped VSV-G-HIV infection of macrophages

Wild type (Wt)-HIV AD8 and BaL strains were available and the NLAD8-VSVG strain was produced by cotransfection with the proviral plasmid in combination with pVSVG. Vesicular stomatitis virus (VSV)-glycoprotein G pseudotyping of envelope defective viruses was performed by cotransfection of 293T cells with a VSV-G expression plasmid (pCMV–VSV-G) at an HIV-AD8/VSV-G plasmid ratio of 10:1. Then, 24 h later, the medium was replaced, and supernatants containing lentiviral particles were collected at 48 and 72 h after transfection, pre-cleared by centrifugation, ultra-concentrated over 5 h at 18,000 rpm; the pellet was resuspended in DMEM supplemented with 10% fetal bovine serum (FBS) and stored at −86°C until use. The amount of HIV-capsid (p24 antigen) in viral stocks was assessed by a commercial ELISA assay (INNOTEST^®^ HIV Antigen mAb). Macrophages were infected with two different inoculums such as 0.01 pg of p24/cell (named “low”) and 1.0 pg of p24/cell (named “high”) with the macrophage-tropic HIV strains AD8 or BaL, as described previously (38). These two HIV strains are replication-competent, macrophage-tropic, CCR5-using, molecular clones of HIV-1. These two viral inoculums correspond to MOI 0.5 and 50, respectively (39), and reflect plausible viral loads that appear at different stages of natural infection. The assay of HIV replication inhibition was performed by treating the MDMs with NVP 1 μM prior to virus infection. Infectivity and replication were assessed by measuring p24 intracellular expression using the KC57 monoclonal antibody labeled with phycoerythrin against p24 (PE-KC57 [FH190-1-1] (6604667) protein (Beckman Coulter, United States) by flow cytometry, and p24 level in cell supernatants by ELISA.

When necessary to inhibit HIV replication, nevirapine as a potent noncompetitive inhibitor of the retroviral enzyme reverse transcriptase (RT) was used. As nevirapine selectively inhibits HIV *in vitro* (IC50 = 40 nM), it was used at 1 μM.

### 2.3 Flow cytometry analysis

Cells detached by Accutase^®^ (StemCell Technologies, United States), were stained for surface antigens for 30 min at 4°C with the following antibodies: APC anti-human CD206 (cat550889), PE anti-human CD80 (cat566992), FITC anti-human HLA DR (cat555560), FITC anti-human CD9 and mouse anti-human CD81 primary antibody (cat555675) (from BD Biosciences, United States), Alexa Fluor 488 goat anti-mouse IgG H&L secondary antibody (ab150117) and APC Anti-CCR5 antibody (ab176536) (from Abcam, United States). Intracellular staining was performed on fixed and permeabilized cells with Fixation/Permeabilization Kit (Cat554714, BD Biosciences, USA) according to the manufacturer’s instructions for 30 min at 4°C with mouse anti-human Cathepsin K primary antibody (ab37259) and Alexa Fluor 488 goat anti-mouse IgG H&L secondary antibody (both from Abcam, United States). The percentage of cellular death was assessed by staining with APC-conjugated annexin-V and 7-AAD, using the Annexin-V/7-AAD apoptosis detection kit (BD Biosciences, United States). As a positive control of cell death, we have exposed briefly cells to freeze-thaw cycles. Data were acquired using a FACSCanto II (Becton Dickinson, United States) and analyzed with FlowJo.v10.6.2 (Ashland, United States).

### 2.4 Assessment of osteoclast differentiation

Cells were fixed with PFA 4% and stained for evaluating the generation of mature multinucleated osteoclasts using Tartrate Resistant Acid Phosphatase (TRAP) (Sigma-Aldrich, St. Louis, MO, United States) according to the manufacturer’s protocol. Briefly, at the end of the experimental timeline cells were washed twice with 1X PBS and fixed with a fixative solution comprised of citrate, acetone, and 4% formaldehyde for 10 min at 37°C. After washing twice with 1X PBS, fixed cells were stained for TRAP at 37°C in dark for 1 hour. Multinucleated TRAP-positive cells with ≥ 3 nuclei were considered mature osteoclasts. TRAP-positive multinucleated cells were further counted and imaged using an inverted microscope 200x (ECLIPSE, TS100, Nikon).

### 2.5 Measurement of TNF-α, IL-6, and IL-1β concentration

TNF-α, IL-6, and IL-1β were measured by sandwich ELISA in culture supernatants from HIV-infected cells and non-infected controls, using paired cytokine-specific monoclonal antibodies, according to the manufacturer’s instructions (BD Pharmingen, USA).

### 2.6 CCR5 blocking and antagonism experiments

CCR5 blocking was assessed using 1000 ng/mL, 500 ng/mL, and 5 ng/mL of HIV (AD8) recombinant gp120 (CM235) obtained from the NIH AIDS Reagent Repository (Bethesda, MD, United States). CCR5 antagonizing was carried out using 3 μM TAK-779 (Sigma-Aldrich, St. Louis, MO, United States, cat N° 229005-80-5).

### 2.7 Quantitative real-time PCR

Quantitative RT-PCR (qPCR) was used to detect gene expression of key regulators of osteoclast differentiation. Total RNA was then isolated on the twelfth day of culture using a Sigma Genelute RNA isolation kit. RNA was quantified on a nanodrop spectrophotometer, and cDNA was produced using the ImPromII Reverse Transcription System (Promega). Real-time PCR was performed on a StepOne PCR system (Applied Biosystems) using the DNA-binding dye SYBR green for the detection of PCR product. 2 µL of cDNA was added to a final reaction volume of 25 µL containing 0.05 U/µL Taq polymerase, SYBR green, and specific primers (0.2 µM each). The forward and reverse primer sets used for PCR were as follows (5’ to 3’): GAPDH, F: CTCTGACTTCAACAGCGACAC, R: AGCCAAATTCGTTGTCATAC; RANK, F: GGTGGTGTCTGTCAGGGCACG, R: TCTCCCCCACCTCCAGGGGT; DC-STAMP, F: GTTGGCTGCCCTGCACCGAT, R: TCCCTCATCCTGGGGCTGCC; and NFATc1, F: GGTCTCGAACACTCGCTCTGCC, R: GCAGTCGGAGACTCGTCCCTGC. Cycling conditions for GAPDH, DC-STAMP, and NFATc1 were 95°C for 5 min followed by 40 cycles of 95°C for 15 seconds, 55°C for 15 seconds, and 72°C for 30 seconds. Cycling conditions for RANK were 95°C for 5 min followed by 40 cycles of 95°C for 15 seconds, 60°C for 15 seconds, and 72°C for 30 seconds. Melting curve analysis was then performed. All primer sets yielded a single product of the correct size. The fold change (relative expression) in gene expression was calculated using the relative quantification method (2−ΔΔCt) (40). Relative expression levels were normalized against GAPDH. Intra-experiment CT value differences between samples were less than 0.5.

### 2.8 Immunofluorescence microscopy

On day 9 post-seeding cells were fixed with PFA 4% for 10 min at room temperature and were first incubated with mouse anti-human CD81 primary antibody (cat555675, BD Biosciences, United States) diluted in Perm Wash (cat554723, BD Biosciences, United States) for 60 min at 4°C, and then with Alexa Fluor 488 goat anti-mouse IgG H&L secondary antibody (ab150117) for 45 min at 4°C. Afterward, cells were washed and stained with PE-KC57 against p24 protein (cat6604667, Beckman Coulter, United States) for 30 min. DAPI (Invitrogen, Thermo Fisher Scientific, USA) was used for nuclear staining for 15 min at room temperature. Cells were visualized with an ECLIPSE, TS100, Nikon fluorescence microscope, images were processed with Image J software (version 1.47).

### 2.9 Adhesion assay

On day 6 post-seeding, an adhesion assay was performed with infected macrophages then differentiated into osteoclasts and mock-infected controls. The supernatant was removed and cells were washed twice with PBS 1X. Then cells were incubated with Accutase^®^ to detach the cells (StemCell technologies) or PBS for 10 min at 37°C. After incubation, cells were washed twice with PBS, adherent cells were fixed with PFA 4%, and nuclei were stained with DAPI (Invitrogen, Thermo Fisher Scientific, USA). Five images per well were taken (200X) (ECLIPSE, TS100, Nikon), all images were processed and the nuclei were quantified using Image J software (version 1.47).

### 2.10 Assessment of bone resorption

To assess bone resorption activity, macrophages were seeded on bovine cortical bone slices (Boneslices, Inc.) and differentiated into osteoclasts. The dimensions of bone slices are (i) Diameter: 6mm; (ii) Thickness: 0.4mm. Resuspended OC precursors were seeded in 96-well plates on 0.4 mm thick bovine cortical bone slices at a density of 1 × 10^5^ viable cells per bone slice. Following complete cell removal by several washes with water, bone slices were stained with toluidin blue (Sigma-Aldrich) to detect resorption pits under a light microscope (ECLIPSE, TS100, Nikon). The surface of bone degradation areas was quantified manually with ImageJ software (version 1.47).

### 2.11 Statistical analysis

Where applicable, statistical analysis was performed. The exact values of n (donors) can be found in the figure legends. All statistical analyses were performed using GraphPad Prism 7.0 (GraphPad Software Inc., San Diego, CA, USA). The statistical tests were chosen according to the following. Two-tailed paired or unpaired t-test was applied on data sets with a normal distribution (determined using Kolmogorov-Smirnov test), whereas two-tailed Mann-Whitney (unpaired test) or Wilcoxon matched-paired signed rank tests were used otherwise. p < 0.05 was considered as the level of statistical significance (* p ≤ 0.05; ** p ≤ 0.01; *** p ≤ 0.001).

## 3 Results

### 3.1 The HIV replication kinetics during osteoclastogenesis differ according to the inoculum size without modifying cell viability

**Figure 1:**
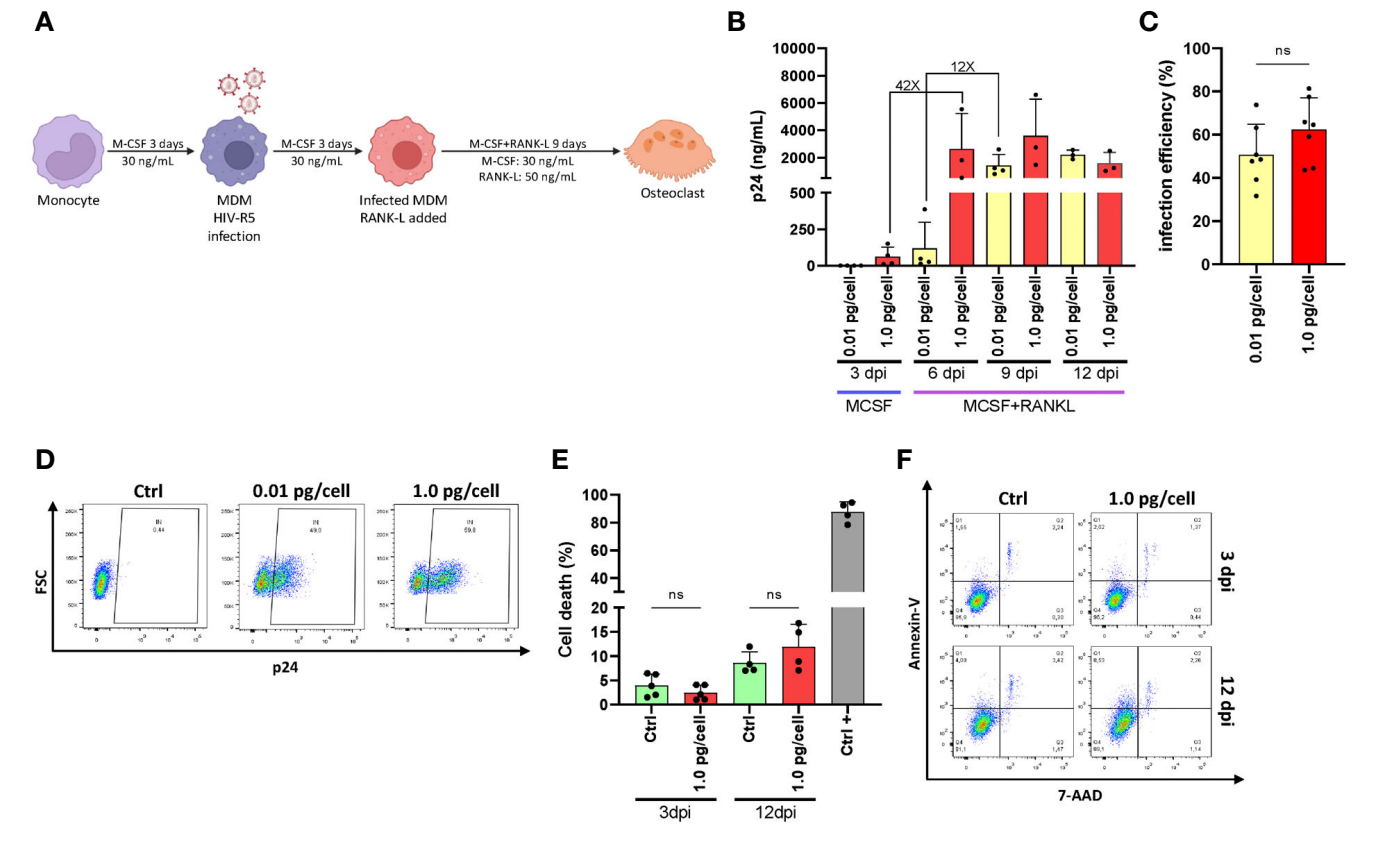
HIV replication kinetics during osteoclastogenesis. **(A)**Representation of the experimental timeline schedule. **(B)** Kinetics of HIV replication using low (0.01 pg/cell) and high (1.0 pg/cell) viral inoculum, measuring p24 antigen in culture supernatants by ELISA during osteoclast formation. **(C)** The efficiency of HIV infection (measured by flow cytometry as a percentage of cells expressing intracellular p24 antigen) at 12 dpi. **(D)** Representative dot plots were obtained at 12 dpi by flow cytometry using KC57 monoclonal antibody against gag p24 in control and HIV-infected cells. **(E)** Measurement of cell death (as a percentage of cells stained with Annexin V/7-AAD) upon HIV infection with high inoculum at two different time points, 3 dpi, and 12 dpi. **(F)** Representative dot plots obtained by flow cytometry measuring Annexin V/7-AAD staining at time points represented in **(E)** Data are expressed as mean ± SD obtained from 4-6 independent experiments performed with cells from different donors. ns, not significant.

**Figure 1A** shows the experimental timeline. After challenging monocyte-derived macrophages (MDM) with two different HIV inoculums, different viral replication kinetics were observed, even during their differentiation into osteoclasts. Thus, the HIV infection efficiency and replication at 3-, 6-, 9-, and 12-days post-infection (dpi) were assessed by measuring the relative abundance of intracellular p24-expressing cells (using flow cytometry) and soluble p24 in cell supernatants (using ELISA, expressed as mean ± SD), respectively. As depicted in **Figure 1B**, an early and massive HIV replication was found after a high inoculum was used (1.0 pg/cell) showing a jump (42-fold increase) in their levels from 3 (62.0 ± 65.3 ng/mL) to 6 dpi (2622.7 ± 2614.8 ng/mL) that was significantly higher than low-inoculum (p=0.008). When viral infection was carried out using a low inoculum (0.01pg/cell), a 12-fold increase occurred between 6 (118.7 ± 178.9 ng/mL) and 9 dpi (1422.2 ± 805.6 ng/mL). As shown in **Figure 1C**, at the end of the experimental timeline (12 dpi), the HIV infection efficiency was similar for both inoculums (62.4 ± 14.7% vs. 50.7 ± 14.1%) in line with viral replication levels (p24 antigen) measured in the cell culture supernatant. As shown in **Figure 1D**, cell death levels did not differ early after infection (4.0 ± 2.3% vs. 2.5 ± 1.5%) or at 12 dpi (11.9 ± 4.7% vs. 8.6 ± 2.3%) even with the higher viral inoculum. Thus, two different HIV replication kinetics were observed early after RANK-L treatment during osteoclast differentiation, according to the initial viral inoculum but without altering the cell viability. Hence, a mild versus a massive proportion of osteoclast precursors were infected using low (0.01 pg/cell) and high (1.0 pg/cell) HIV inoculum, respectively.

### 3.2 The viral inoculum, but not the HIV R5-tropic strain infecting macrophages, affects osteoclast formation

**Figure 2:**
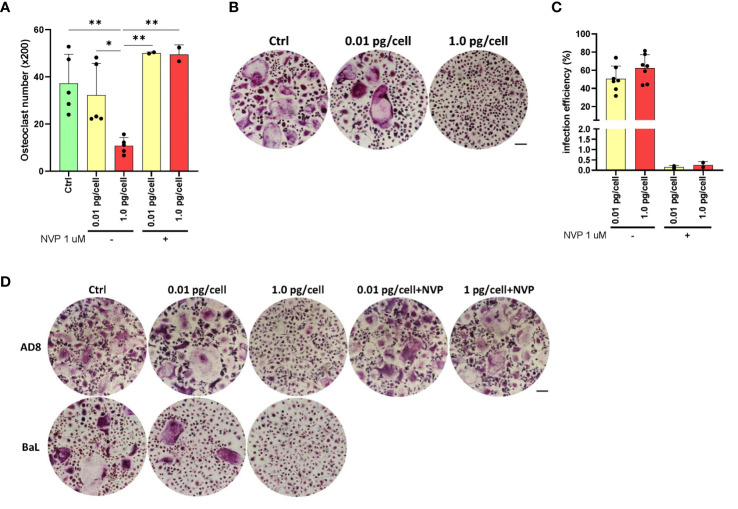
HIV R5-tropic strain modulates osteoclast formation. Quantification of the number of TRAP-positive osteoclasts after culturing for 12 days and the number of TRAP-positive osteoclasts obtained from monocytes after 15 days of incubation with M-CSF, and 9 days with RANK-L **(A)**. Representative images of A (x200) **(B)**. Measurement of HIV infection efficiency (as a percentage of cells expressing p24-capsid antigen measured by flow cytometry using PE-KC57) at 12-dpi for each inoculum in the absence or presence of the reverse transcriptase inhibitor nevirapine (1μM) **(C)**. Morphology of TRAP-positive osteoclast formation in cell cultures at 12 dpi, using the two HIV inoculums (low and high), and two different R5-tropic HIV strains (AD8, and BaL), in the presence or absence of nevirapine (only for the AD8 strain) (x200) **(D)**. Scale bar: 200 µm. Data are expressed as mean ± SD obtained from 4-6 independent experiments performed with cells from different donors. *p < 0.05, **p < 0.01.

To determine whether HIV-infected MDM acquires osteoclast characteristics, human primary macrophages derived from primary monocytes (from 4-5 donors) were infected with two HIV R5-tropic strains, NLAD8 and BaL. Twelve days post-infection, osteoclast formation in culture was quantified by manually counting TRAP-positive, multinucleated (≥3 nuclei) cells visualized under the microscope (x200; as mean number ± SD). As shown in **Figures 2A, B**, HIV infection of MDM with a low inoculum of either NLAD8 or BaL R5-tropic strains triggered similar MDM fusion into osteoclasts compared to non-infected cells (control: 37.2 ± 12.4; HIV-low: 32.3 ± 13.3). In contrast, osteoclast formation was significantly lower when MDM was infected with a high HIV inoculum (10.8 ± 3.4). To elucidate the massive HIV-replication impact on osteoclast formation, it was inhibited using nevirapine (NVP), a non-nucleoside reverse transcriptase inhibitor. As shown in **Figure 2C**, HIV infection efficiency measured at 12 dpi diminished drastically in cultured macrophages treated with nevirapine 1 µM prior to being challenged with HIV (from 51.8 ± 19.9% to 0.3 ± 0.2% and from 31.4 ± 28.5% to 0.2 ± 0.1% for high and low-viral inoculum, respectively). As shown in **Figure 2D**, osteoclast formation from NVP-treated cultured cells appeared unchanged even with macrophages exposed to a high inoculum (50.1 ± 0.4 and 49.5 ± 4.1). Thus, these results indicate that osteoclast formation is strongly impaired when its precursors are massively and productively HIV-infected.

### 3.3 Analysis of the macrophage activation profile and cytokines released after HIV infection with low and high viral inoculums

**Figure 3:**
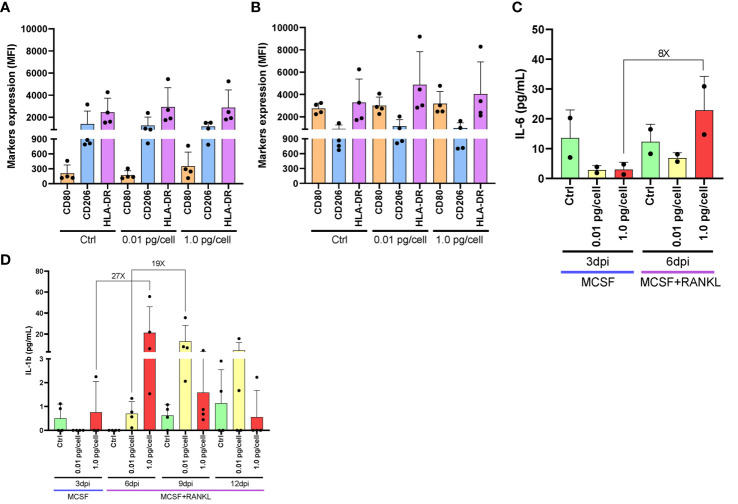
Macrophages activation profile and cytokines released after HIV infection. Surface marker expression and cytokine secretion during HIV infection of monocyte-derived macrophages. Surface marker expression (CD80, CD206, and HLA-DR) was determined by flow cytometry in cultured monocyte-derived macrophages after 6 days with M-CSF and 3 days post-inoculation (dpi) using the two HIV inoculums, in the absence of LPS stimulus **(A)**. Surface marker expression was determined in the presence of LPS stimulus **(B)**. IL-6 secretion was measured by ELISA at different times during the osteoclast differentiation process after HIV infection with each viral inoculum, both prior to and following RANK-L addition. The internal bracket depicts the fold change **(C)**. IL-1b secretion was measured by ELISA at different times during the osteoclast differentiation process after HIV infection with each viral inoculum, both prior to and following RANK-L addition, throughout the experimental timeline **(D)**. Data are expressed as mean ± SD for 2 to 4 independent experiments. MFI, Mean Fluorescence Intensity.

Considering their dependence on the surrounding environment, the activation profile of macrophages was analyzed after their exposure to HIV-low and high inoculum. According to the surface markers expression (CD206+/HLA-DR+/CD80-) of both non-infected control and HIV-infected macrophages, macrophages differentiated with M-CSF for 6 days exhibit an M2-like profile as shown in **Figures 3A, B**, regardless of the viral inoculum size. These M2-like macrophages shifted toward an M1 activation profile (CD206+/-/CD80+) when stimulated with lipopolysaccharide (LPS, 100 ng/mL) for control and both viral inoculums as well. Afterward, when the level of M1-associated cytokines (tumor necrosis factor-α -TNF-α-, interleukin-6 -IL-6-, and IL-1β) was measured, TNF-α was undetectable for control and both viral inoculums. **Figure 3C** shows an 8-fold increase between 3 and 6 dpi in the IL-6 level among osteoclasts precursors infected with the high viral inoculum (2.9 ± 1.4 and 22.8 ± 11.4 pg/mL). In contrast, the IL-6 change was significantly lesser (2-fold) by those cells infected with the small inoculum (3.1 ± 2.4 and 6.9 ± 1.8 pg/mL). Likewise, as shown in **Figure 3D**, the amount of IL-1β released accompanied the HIV replication kinetics followed for each condition. When a high HIV inoculum was used, the IL-1β level jump to a maximum level earlier and concurrently with RANK-L addition (from 0.8 ± 1.3 to 21.3 ± 24.5 pg/mL: 27-fold change) that was significantly higher than low-inoculum (p=0.03). A similar IL-1β jump level but three days later was released when the low-HIV inoculum was used (from 0.7 ± 0.5 to 13.1 ± 15.2 pg/mL: 19-fold change). Thus, even in the presence of an M2-like profile, changes in the timing but not the amount of IL-1β release are seen in response to the size of the HIV inoculum during osteoclastogenesis.

### 3.4 The CCR5 expression in osteoclast precursors is directly influenced by the HIV-inoculum size and its blocking, antagonizing, or even bypassing impairs the osteoclastogenesis

**Figure 4:**
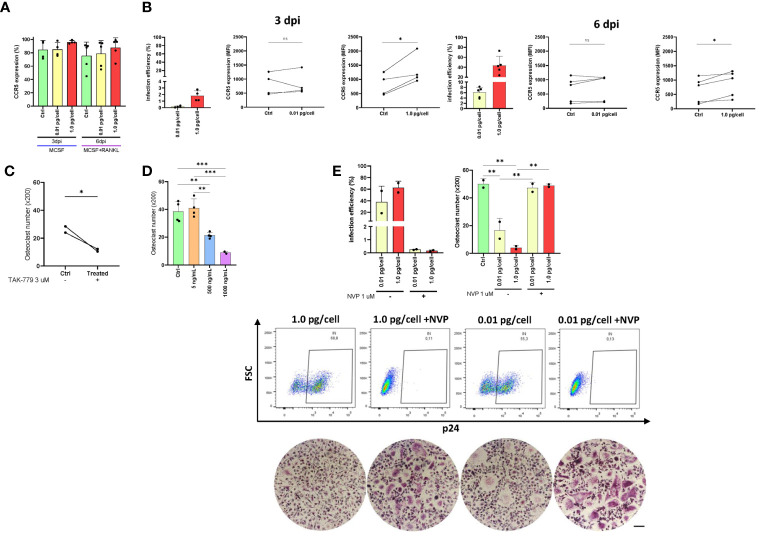
CCR5 expression in osteoclast precursors. Expression of CCR5 on the cell surface (as a percentage of positive cells) in osteoclast precursors after HIV infection (using both inoculums, 0.01 and 1.0 pg/cell) at 3 and 6 dpi measured by flow cytometry **(A)**. The efficiency of HIV infection (measured by flow cytometry as the percentage of cells expressing intracellular p24 antigen) and change in CCR5 expression level (as MFI) between paired samples (control vs. HIV infected) in osteoclast precursors infected with each HIV inoculum at 3 dpi and 6 dpi **(B)**. Quantification of the number of TRAP-positive osteoclasts after CCR5 antagonism using TAK-779 **(C)** or CCR5 blocking using three different concentrations of recombinant HIV (AD8)-gp120 **(D)**. The efficiency of VSV-G-pseudotyped HIV infection (measured by flow cytometry as the percentage of cells expressing intracellular p24 antigen) and the number of TRAP-positive osteoclasts (x200) at 12 dpi using the two viral inoculums in the absence and presence of nevirapine **(E)**. Scale bar: 200 μm. Data are expressed as mean ± SD obtained from 2-4 independent experiments performed with cells from different donors. *p < 0.001, **p < 0.001, and ***p < 0.0001. ns, not significant.

The CCR5 plays a dual role as an HIV co-receptor as well as an osteoclastogenesis regulator. Here, we evaluated CCR5 expression levels in osteoclast precursors after their exposure to both viral inoculums. As shown in **Figure 4A**, the relative abundance (percentage) of cells expressing CCR5 at 3 dpi and 6 dpi were slightly greater when the high inoculum was used than the low-inoculum and non-infected control. However, as displayed in **Figure 4B**, the CCR5 expression level (as median fluorescence intensity –MFI-) increased significantly at 3 and 6 dpi using 1 pg/cell but not when the low inoculum was used (and non-infected control). The HIV infection efficiencies (% of cells expressing HIV-p24 antigen) at 3 dpi were 0.2±0.1% and 1.8±0.8% using low and high-vial inoculum, respectively. Such efficiencies at 6 dpi were 6.1±2.0% and 43.8±18.1%. Subsequently, CCR5 expression level among cells exposed to high-inoculum decreased significantly (p<0.05) from 3 dpi to 6 dpi after RANK-L addition (1614.4±796.1 vs. 876.0±471.7).

As exposure to a higher inoculum implies greater simultaneous co-receptor occupancy, we performed CCR5 blocking (using a recombinant gp120 from AD8) and CCR5 antagonism (using TAK-779 3 μM, a potent and selective non-peptide antagonist) experiments. As shown in **Figures 4C, D**, both treatments decreased significantly osteoclastogenesis in a dose-dependent manner. When the CCR5 was antagonized with TAK-779, the mean number of osteoclasts ( ± SD) diminished from 26.3 ( ± 3.2) to 11.4 ( ± 1.2) whereas the CCR5 blocking using gp120 (500 and 1000 ng/mL) decreased from 38.5 ( ± 7.4) to 21.3 ( ± 2.2) and 8.9 ( ± 1.0) osteoclasts (x200). Thus, both HIV-gp120 blocking and TAK-779-antagonizing of CCR5 interfere the osteoclastogenesis.

We next investigated whether infection with an HIV-R5 virus pseudotyped with the G glycoprotein of VSV (lacking gp120 thus unable to use CCR5 for entry) affected osteoclastogenesis. As shown in **Figure 4E**, the efficiency of infection with HIV-VSV was high at 12 dpi (62.7 ± 15.2%) irrespectively of the inoculum of pseudotyped virus used, resembling those achieved with the higher inoculum of the R5-wt viruses. In both conditions at 12 dpi, the number of osteoclasts (x200) significantly decreased from 50.1 ± 3.8 (non-infected control) to 16.7 ± 8.6 and 4.1 ± 1.6 using low and high inoculum, respectively. However, when viral replication was inhibited with NVP, osteoclastogenesis was recovered (47.3 ± 4.1 and 48.9 ± 1.3).

These findings demonstrate that both a CCR5-dependent (by its occupancy) and a CCR5-independent form of osteoclast differentiation impairment occurs during HIV productive infection in macrophages.

### 3.5 RANK, NFATc1, and DC-STAMP mRNA levels are differently regulated during osteoclastogenesis according to the HIV inoculum size

**Figure 5:**
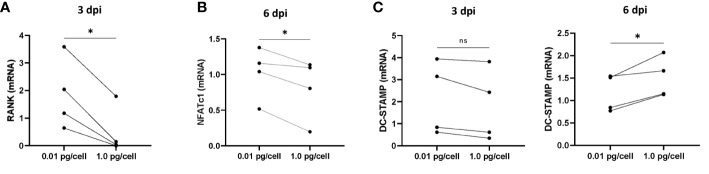
mRNA levels of RANK, NFATc1, and DC-STAMP during osteoclastogenesis after HIV infection. The mRNA levels of RANK **(A)**, NFATc1 **(B)**, and DC-STAMP **(C)** were quantified using real-time quantitative PCR (RQ-PCR) in paired osteoclast precursor samples obtained from the same donor that were infected using two HIV inoculum and measured at 3 days post-infection (dpi) and/or 6 dpi, as indicated. The values of mRNA levels are relative to GAPDH (a housekeeping gene). *p< 0.001. ns, not significant.

Considering that in both physiological and pathological processes, the receptor activator of nuclear factor kappa B ligand (RANK-L), and its receptor, RANK play critical roles in controlling the development, activation, and survival of osteoclasts, we have measured the mRNA levels of RANK in macrophages after HIV infection (3 dpi) with low and high-inoculum (values normalized with GAPDH as housekeeping gene). As shown in **Figure 5A**, the RANK mRNA level in macrophages remained unchanged using the low HIV inoculum but it diminished significantly after the high-inoculum challenge.

According to the timeline, at 3 dpi the macrophages are exposed to RANK-L activating other signals involved in differentiation and fusion. In particular, NFATc1 is a master transcription regulator of osteoclast differentiation. As the RANK/RANK-L interaction triggers NFATc1 transcription, we have measured its transcriptional level post-RANK-L stimulus. As presented in **Figure 5B**, the mRNA levels in osteoclasts precursors exposed to a high inoculum of HIV were significantly dropped than those control or exposed to a low inoculum.

Concomitantly, we investigated the mRNA levels of DC-STAMP, a surface receptor required for osteoclast precursors fusion. As shown in **Figure 5C**, the DC-STAMP mRNA levels remained unaltered prior to RANK-L exposure irrespective of the HIV-inoculum size but it was significantly increased in cells exposed to higher HIV inoculum.

Thus, the magnitude of the HIV infection in osteoclast precursors may affect the transcriptional level of key regulators of osteoclastogenesis interfering with their fusion and differentiation.

### 3.6 The expression of tetraspanins CD9 and CD81 is influenced during the osteoclast differentiation and by the HIV-inoculum size

**Figure 6:**
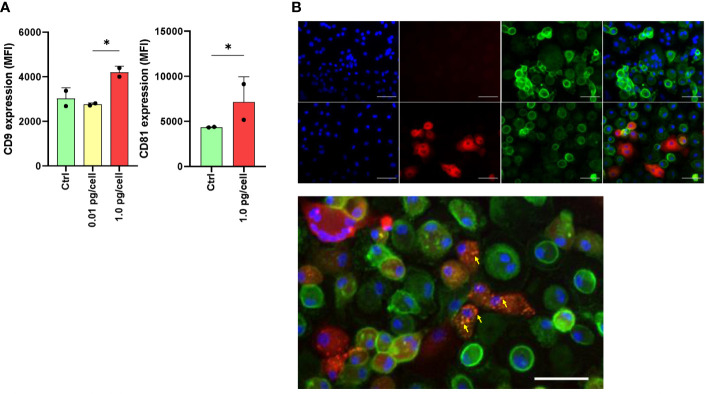
Expression of tetraspanins CD9 and CD81 during osteoclast differentiation and HIV infection. The expression level of CD9 and CD81 was measured using flow cytometry (mean fluorescence intensity per cell, or MFI) at 6 days post-infection (dpi) using both HIV inoculums. The results are representative of two independent experiments performed using cells from different donors **(A)**. Osteoclasts and their precursors at 6 dpi of HIV infection with the low-inoculum (upper panel) and control (non-infected) (lower panel) were stained with DAPI (for cell nuclei), PE (for HIV-p24 capsid antigen), FITC (for CD81), and merged using immunofluorescence staining and deconvolution microscopy to examine changes in the cellular localization of tetraspanins during maturation and HIV infection (x400) **(B)**. Osteoclasts and their precursors at 6 dpi of HIV infection with the low-inoculum showing CD81 expression (green) and HIV-infected giant multinuclear cells (red, with nuclei in blue). **(C)** Yellow arrows show perinuclear structures resembling viral-containing compartments (VCC). Scale bars: 20 μm. *<0.05 indicates a statistically significant difference.

As HIV entry and egress are influenced by tetraspanin microdomains, we investigated the effect of HIV infection on CD9 and CD81 expression and its repercussions in the fusion process during osteoclastogenesis. As shown in **Figure 6A**, among macrophages infected with a high HIV inoculum, the expression of CD9 and CD81 at 6 dpi, measured as MFI by flow cytometry, was significantly higher than in control cells or cells exposed to a low viral inoculum as follows: 4196.0 ± 277.2 vs. 2760.0 ± 63.6, and 7145.5 ± 2800.8 vs. 4354.5 ± 43.1, respectively.

After 6 days of osteoclast formation in non-infected control cells, we observed a lower expression of CD81 on the membranes of multinucleated giant cells compared to mononuclear cells. This difference was also observed in multinucleated giant cells infected with HIV (p24 positive) after exposure to a high-viral inoculum. Furthermore, as shown in **Figures 6B, C**, at 6 dpi using double immunostaining for CD81 in HIV-infected cells (p24-positive cells), CD81 expression appeared to relocate to perinuclear structures resembling viral-containing compartments (VCCs) (**Figure 6C**).

### 3.7 Osteoclasts and macrophages adherence is differentially impaired according to the HIV inoculum size

**Figure 7:**
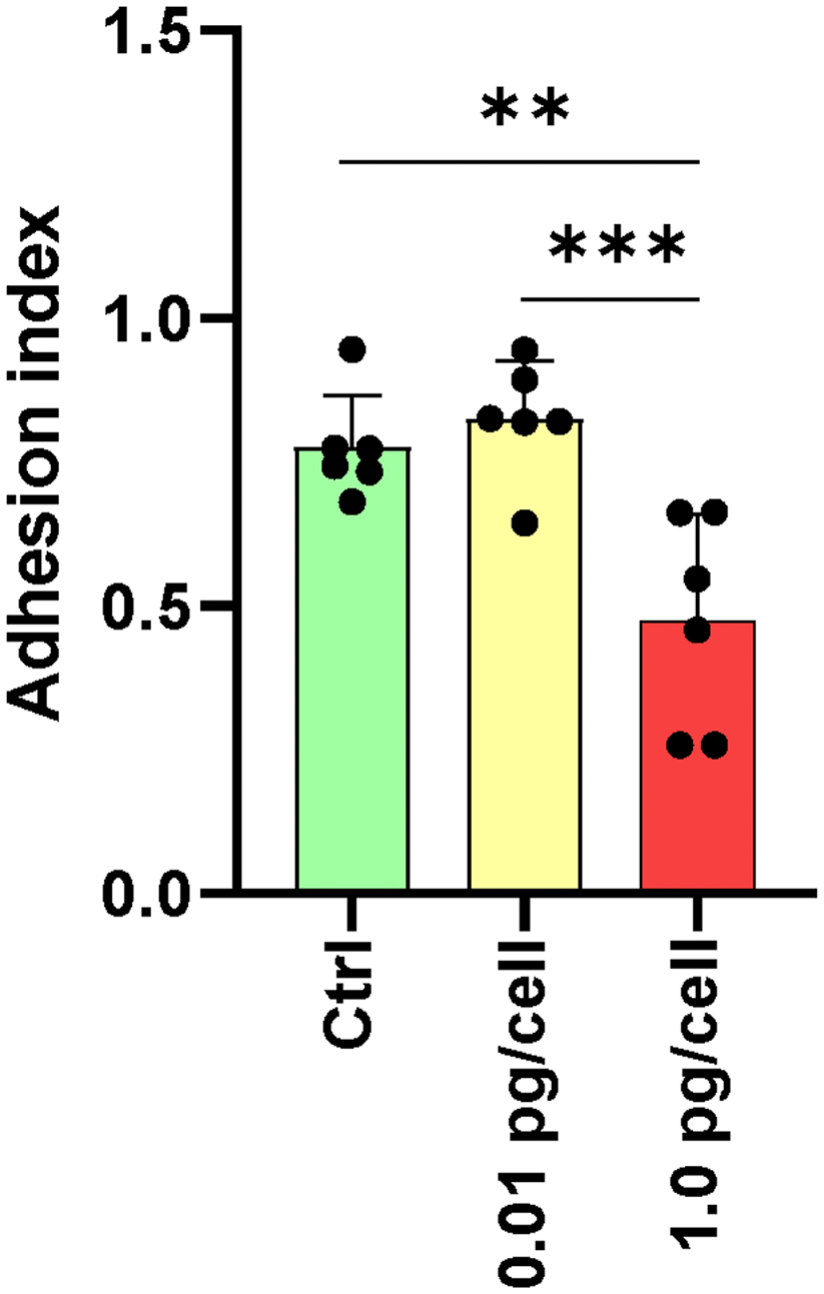
Adhesion of osteoclasts and their precursors. The adhesive properties of osteoclasts vary with the size of the HIV infection inoculum after 10 days of differentiation. The graph represents the average of five fields per condition from a total of six donors. **p ≤ 0.01 and ***p ≤ 0.001 indicate statistically significant differences.

The osteoclasts attachment/detachment was assessed as a critical factor for bone degradation. When infected macrophages were exposed to a low HIV inoculum, osteoclasts precursors were more resistant to detachment induced by Accutase® treatment than massively HIV-infected counterparts (**Figure 7**). HIV infection with low-inoculum (0.01 pg/cell) retained the adhesive properties of osteoclasts but when the HIV inoculum was high (1 pg/cell), the cell adherence was significantly lesser (0.5 ± 0.2) than non-infected (0.8 ± 0.1) and infected with low-HIV inoculum (0.9 ± 0.1). At 3 dpi, non-infected and HIV-infected osteoclast precursor cells were detached using Accutase® for 10 minutes, and the percentage of remaining adherent cells was quantified by counting nuclei (adhesion index). Due to the reduced adhesion caused by the high HIV inoculum, osteoclast mobility on bone is likely slowed, which should be a factor in the altered architecture of resorption lacunae and the decreased bone destruction activity.

### 3.8 Osteoclasts’ cathepsin K expression and resorptive ability are modulated by HIV infection

**Figure 8:**
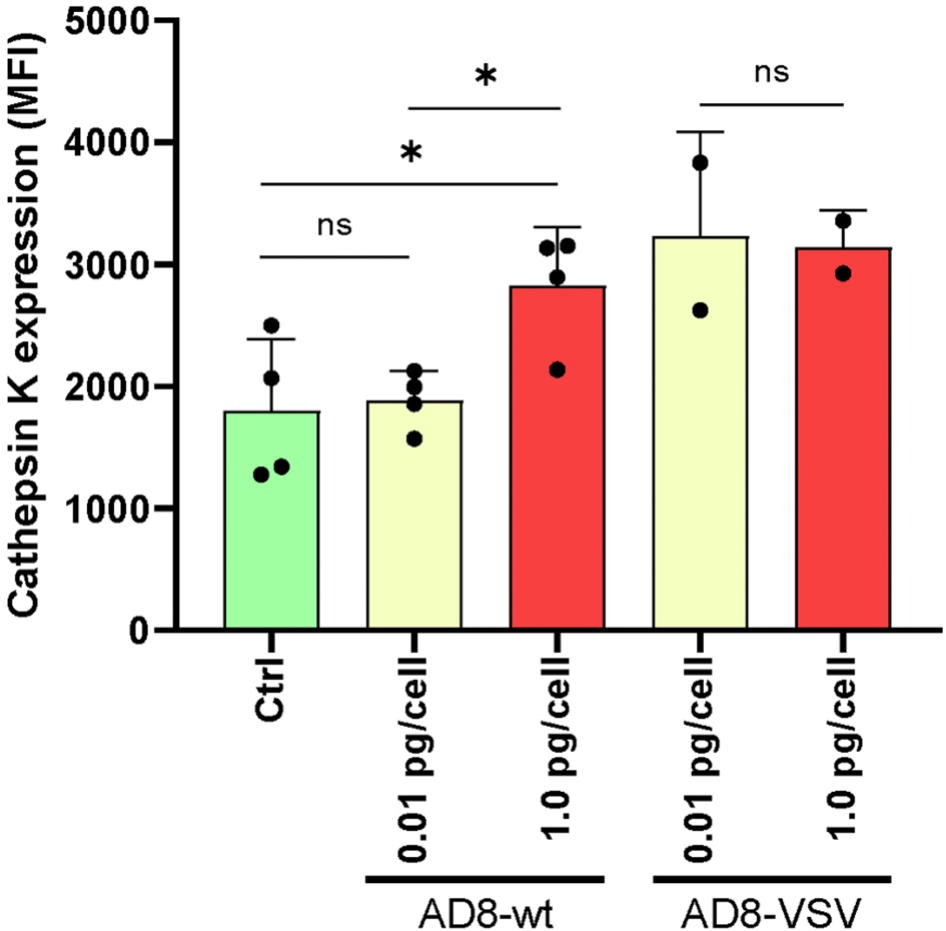
Cathepsin K expression in osteoclasts and precursors after HIV infection. The level of cellular expression was measured using flow cytometry at 12 days post-infection (dpi) with two different viruses: wild-type HIV (AD8) and pseudotyped HIV (AD8)-VSV, each at two different inoculum levels (low and high). Mean fluorescence intensity (MFI) values are expressed as mean ± SD obtained from independent experiments performed using cells from 2-4 different donors. *p < 0.05 indicates a statistically significant difference, while “ns” indicates that the difference is not significant.

The enzyme cathepsin K (CTSK) first discovered in differentiated osteoclasts plays a critical role in degrading the bone matrix and contributes to osteoclast-mediated bone resorption. To evaluate the functional impact of HIV infection on the differentiated osteoclast, we have measured cellular CTSK expression level (as mean fluorescence intensity –MFI-) using flow cytometry at 12 dpi. For this goal, both R5 HIV-wt and pseudotyped HIV-VSV were used (each one with low and high inoculum). As shown in **Figure 8**, cells exposed to a high-HIV wt inoculum depicted a significantly higher CTSK level than low-inoculum and non-infected control. When using pseudotyped HIV, a high efficiency of infection at 12 dpi was observed irrespectively of the HIV-VSV inoculum accompanied in both for a high level of CTSK expression.

**Figure 9:**
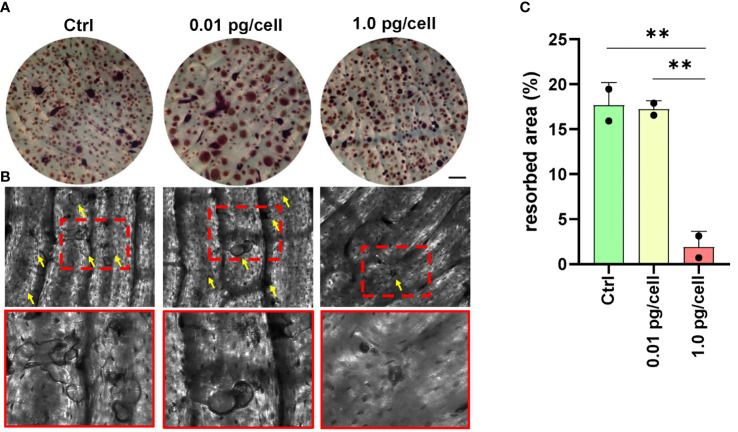
Bone resorption by osteoclasts. Representative data from a donor were obtained using a light microscope to show TRAP-positive (red) osteoclasts differentiated from macrophages during 12 days of culture with RANK-L and M-CSF on bone slices obtained from control (non-infected precursors) and HIV-infected precursors exposed to different viral inoculum **(A)**. The bone resorption pits formed by osteoclasts from precursors (control, and HIV-infected using high and low-inoculum) cultured for 9 days were visualized by staining the slices with toluidine blue. Zoomed-in views of red dashed squares provide more detailed observation **(B)**. The resorption area was observed under a light microscope (x100) and analyzed using Image-J **(C)**. Scale bar: 100 μm. The data represent the mean ± SD of two independent donors, and **p < 0.01 indicates a statistically significant difference.

To determine if HIV-induced impairment in osteoclastogenesis affected resorptive ability, osteoclast precursors were cultured on bone slices (non-infected, and HIV-infected with low and high viral inoculum) and then differentiated into mature osteoclasts by RANK-L for 9 days. As shown in **Figure 9**, the presence of many resorption pits (discriminated from the gray background by an intensely dark stain) was observed in both non-infected and low-HIV inoculum conditions, but the high HIV inoculum strongly reduced the resorption area. The aforementioned findings indicate that the OC traits such multinuclearity and bone resorption competence are modulated according to the HIV inoculum size that challenges the osteoclast precursors.

## 4 Discussion

The dysregulation of the bone remodeling system, which involves the interaction between osteoblasts and osteoclasts, can result in osteolytic bone disease, which is a common complication of HIV infection. Several studies have reported that HIV infection contributes to the emergence of osteolytic illness (41–44). For instance, HIV-infected human macrophages secrete RANK-L, which enhances osteoclast recruitment (45). Additionally, HIV targets the bone degradation machinery of osteoclasts, leading to bone loss (26). Furthermore, HIV can replicate in human osteoclasts and enhance their differentiation *in vitro* (24). However, the connection between osteolytic disease and HIV infection remains unclear. Our study demonstrates that a massive HIV infection of primary myeloid-lineage osteoclast precursors (monocyte-derived macrophages) damages their differentiation, adhesion, and osteolytic function without inducing marked cell death. Within 3 to 6 days of challenging them with a high viral inoculum, HIV replication burst in macrophages was rapid and massive, and this level was maintained, severely affecting the formation of osteoclasts. In contrast, when infection was performed with an inoculum 100 times lower, the replication kinetics and burst appeared delayed during the 9-day RANK-L induced differentiation into osteoclasts, which was not qualitatively or quantitatively affected despite achieving similar HIV infection efficiency levels. The different kinetics associated with the viral inoculum size could be observed with at least two different laboratory R5-tropic isolates (AD8 and BaL).

In our experimental timeline, the rapid and massive infection achieved with the higher inoculum occurred immediately prior to the onset of RANK-L-induced osteoclast differentiation. Such high infection efficiency achieved among osteoclast precursors may explain the discrepancies with other studies that used transgenic rats (27) or, MDM infected with a viral inoculum even smaller than our low one (45). It is known that RANK-L reduces cellular expression levels of CCR5 (46) and consequently, it could negatively affect the infection with the lower inoculum, delaying replication. The differentiation of macrophages by M-CSF led them towards an M2-like activation profile that was preserved even after HIV infection. However, the increase in IL-6 and IL-1β levels was earlier and significantly higher when preosteoclasts were infected with the higher inoculum. This elevated IL-6 level may suppress osteoclast progenitors’ differentiation by inhibiting directly RANK signaling pathways (47). The effect of IL-1β on osteoclastogenesis is highly time-dependent. Here, its elevation accompanied the viral replication kinetics according to the viral inoculum, reaching its maximum level concomitantly with the addition of RANK-L when the infection was done with the higher inoculum. Exposure to high levels of IL-1β before or concurrently with RANK-L reduces osteoclast development (48, 49). In contrast, a later peak of IL-1β after RANK-L therapy, as occurred when using the low HIV inoculum, has the opposite effect, promoting the growth and activity of osteoclasts. IL-1 receptors (IL-1Rs) and Toll-like receptors (TLRs) share a cytosolic Toll-IL-1R domain and common intracellular signaling molecules. This phenomenon can be explained by the fact that IL-1β directly inhibits the early stages of human osteoclast maturation by disabling the M-CSF receptor, c-Fms, which is necessary for RANK synthesis after binding with TLRs (49).

In line with this observation, we found that mRNA levels of RANK were significantly downregulated in macrophages massively infected with the high HIV inoculum, impairing RANK-L-induced osteoclast differentiation. Furthermore, the mRNA level of nuclear factor-activated T cells c1 (NFATc1) was also decreased in this condition. The NFATc1 protein is a master regulator of osteoclast differentiation that controls a number of osteoclast-specific genes, such as TRAP and cathepsin K, which could act as a transcriptional factor during HIV replication (50, 51). Besides, we have found higher expression of cathepsin K among cultured cells previously exposed to a high-HIV inoculum than in non-infected or infected with low-viral input, probably reflecting that the enzyme is predominantly retained in mononuclear cells but is not secreted to the extracellular media by osteoclasts. Furthermore, a reciprocal regulation between NFATc1 and the master fusogen, dendritic cell-specific transmembrane protein (DC-STAMP), has been described (52). In this context, Chiu et al. confirmed the presence of surface DC-STAMP in freshly isolated monocytes purified from human peripheral blood mononuclear cells. However, the expression level of DC-STAMP was found to be downregulated in mature osteoclasts. Consistent with this, we observed significantly higher DC-STAMP mRNA levels during impaired osteoclastogenesis in high HIV-inoculum-infected cells when exposed to RANK-L.

Massive HIV infection in osteoclast precursors alters the expression of surface markers involved in their fusion and differentiation, including CCR5 and tetraspanins CD9 and CD81. In macrophages, CCR5 expression is upregulated by macrophage colony-stimulating factor (M-CSF) (53), but it is downregulated by RANK-L (46). We found that prior to the addition of RANK-L, macrophages exposed to a high HIV inoculum expressed significantly higher levels of CCR5 than non-infected controls or low-inoculum. However, upon adding RANK-L, its expression diminished, likely due to an IL-1β peak, which can trigger p53-mediated down-modulation of CCR5, limiting HIV entry in macrophages as well (54). During osteoclast differentiation, their precursors exposed to a high viral inoculum exhibited decreased CCR5 expression. This correlates with our observations of the affected bone-resorption activity of osteoclasts, and their weak adherence (35). Similarly, we observed inhibition of osteoclast formation when CCR5 was chemically antagonized in a dose-dependent manner using TAK-779, as well as after blocking it by exposure to recombinant HIV (AD8)-gp120. Furthermore, we found that such an inhibitory effect was present when osteoclast precursors were infected with pseudotyped HIV-VSV, which replicates profusely after cellular entry in a CCR5-independent manner. Thus, the inhibition of osteoclastogenesis that occurs during massive HIV infection in their precursors involves CCR5 occupancy and the burst of viral replication.

Tetraspanins, such as CD9 and CD81, are a family of integral glycoproteins that span the membrane four times and create specific microdomains based on non-covalent protein-protein interactions. It is known that tetraspanin-enriched microdomains containing CD9 and CD81 suppress HIV viral entry and serve as anchor sites for HIV progeny assembly. These tetraspanins influence cellular permissiveness to viral entry by interacting and altering the localization and accessibility of the CD4 receptor (55). Here, we have observed a significant increase in CD9/CD81 expression during osteoclastogenesis among precursors exposed to a high-HIV inoculum, which may be linked with the essential role of these two tetraspanins in preventing the fusion of mononuclear phagocytes (56, 57). Among osteoclasts, control, and HIV-infected ones, we observed a lower cell surface expression of CD81. Thus, when osteoclast precursors are massively infected, CD9 and CD81 appear to be overexpressed, leading to the inhibition of fusion.

In conclusion, we demonstrate that the magnitude and kinetics of HIV infection in macrophages as precursors of osteoclasts, modulate multiple cellular factors involved in osteoclastogenesis. This knowledge contributes to the development of new strategies to prevent or treat bone disorders in people with HIV.

## Data availability statement

The raw data supporting the conclusions of this article will be made available by the authors, without undue reservation.

## Author contributions

FS performed the experiments and analyzed the data. PJ helped with cell culture. CC helped with the set-up of RT-qPCR. MO and AA helped with the set-up of CD9 and CD81 measurement. RF, AG, and CL helped with viral stocks and culture media preparation. JQ wrote the manuscript. JQ and M.VD designed the experiments, revised the manuscript, and obtained research funding. All authors have read and agreed to the published version of the manuscript.
